# A registry-based modified IDEAL stage 2b evaluation of urological procedures performed using the versius robotic surgical system

**DOI:** 10.1007/s11701-025-02324-2

**Published:** 2025-05-26

**Authors:** Ata Jaffer, Daniel Chia, Sonpreet Rai, Sachin Veer, Kevin Gallagher, Mohammed Aldiwani, Muddassar Hussain, Sohail Nakhuda, Farleigh Reeves, Abhay Rane, Sebastian Ourselin, Alan McNeill, Ben Challacombe, Prokar Dasgupta

**Affiliations:** 1 Guy’s and St Thomas’ Hospital, London, UK; 2 Milton Keynes University Hospital, Milton Keynes, UK; 3 East Surrey Hospital, Redhill, UK; 4 Western General Hospital, Edinburgh, UK; 5 Frimley Park Hospital, Frimley, UK; 6 Stepping Hill Hospital, Manchester, UK; 7 King’s College London, King’s Health Partners, London, UK

**Keywords:** Urological surgery, Versius robotic surgical system, Surgical outcomes, IDEAL

## Abstract

The IDEAL stage 1/2a study demonstrated feasibility of the use of the Versius robotic platform at a single institution. This multi-center collaborative modified stage 2b study adds wider exploration assessing surgical, oncologic and functional outcomes in patients undergoing urological procedures with the Versius. Data were collected from a prospective registry. Surgical outcomes were cross-checked from electronic patient records held at each hospital included in the study. All UK-based centers performing urological procedures using the Versius robot. All patients who underwent a robotic procedure using the Versius robot since inception to Jan 2023. Five UK hospitals have undertaken a total of 70 urological procedures using the Versius (Table 1). Device failure occurred in one of the cases requiring conversion to laparoscopy and a further case required conversion to open due to intra-operative complication (2/70, 2.9%). One positive margin was observed in a robotic prostatectomy patient (12.5%). Immediate continence was achieved in 4 of 8 (50%) after prostatectomy. Eight patients (11.4%) had complications within 30 days, and three were Clavien–Dindo class 3 and one class 4. A range of urological procedures were shown to be feasible and safe using the Versius across multiple sites.

## Introduction

Robotic surgery has evolved significantly over the past 2 decades. Since FDA approval of the Da Vinci robot (Intuitive Surgical, CA) in 2000, newer robotic devices have emerged onto the market [1, 2]. One such robot is the Versius Robotic Surgical System (RSS; Cambridge Medical Robotics Surgical, Cambridge, UK). This was the first commercially available UK-based robotic platform which consists of a modular design with an open console with 3–4 individual bedside units that control 5 mm, wristed robotic instruments. It’s application has expanded over several surgical disciplines following its first clinical in-human analysis in 2019 [3–6].

There are inherent risks in the implementation of innovative surgical devices. These include unexpected complications, longer operation times, the risks associated with the learning curve for new techniques and the occasional requirement for a more traditional technique with proven efficacy when unexpected factors necessitate conversion to another technique. It is therefore important that all new surgical devices undergo appropriate assessment and evaluation which is the basis of the IDEAL framework [7, 8]. The IDEAL framework consists of five key stages: stage 1 (Idea) is composed of early stage development and tends to involve laboratory testing or cadaveric studies. Stage 2a (Development) introduces the new technique to a limited number of patients and modifications are made to refine the procedure. Stage 2b (Exploration) is when the technique is performed in a wider cohort of patients, usually across multiple centers, by a number of different operators. Stage 3 (Assessment) is when comparative studies are initiated to assess efficacy and safety against techniques already in use. This includes randomized controlled trials. Stage 4 (Long-term study) is the final stage and involves monitoring long term safety, assessing real-world effectiveness outside of clinical trials and to evaluate cost-effectiveness [8].

Reeves et al. performed the first IDEAL stage 1/2a study assessing the Versius RSS for a range of urological procedures including radical prostatectomy, nephrectomy, pyeloplasty and adrenalectomy [9]. Although mean docking and console times were high, this study demonstrated that urological robotic procedures were feasible and performed safely. Similarly, Rocco et al. outlined the first detailed description of performing a robotic-assisted radical prostatectomy (RALP) demonstrating feasibility [10]. This modified IDEAL stage 2b study is an extension of the work done by Reeves et al. Here we combine data from all centers performing urological procedures with the Versius RSS in the UK to add further exploration into safety profile and efficacy of the device when compared to best current practice.

## Methods

This was a prospective cross-sectional observational study. The Versius robotic surgical system holds a prospectively maintained data registry of all surgical cases performed with the device. Using this data registry, we were able to identify dates and locations of all procedures performed. All 5 UK hospitals performing urological procedures with the device were approached and agreed to supply additional clinical data with regard to the cases.

## Results

A total of 70 urological procedures using the Versius were performed across the five hospitals. There were 8 robotic prostatectomies (RARP), 39 radical nephrectomies, 4 partial nephrectomies, 8 nephro-ureterectomies, 10 pyeloplasty’s, and 1 adrenalectomy. The median age was 66.75 years, with a median BMI of 25.7.

Median console time was 167.5 min, with a median length of stay of 2 days. There were two intra-operative complications (2/70–2.9%); a device failure in one of the RARP cases requiring conversion to laparoscopy and a splenic bleed during a robotic nephrectomy which required conversion to an open technique (Table 1).

**Table 1:** Main results

Procedure	No. of cases	Median age	Median BMI	Median console time (min)	Intra-op complications	Blood transfusion	Positive margin	Median length of stay (days)	30-day complication
Robotic Prostatectomy	8	67.5	26.5	257.5	1/8	0/8	1/8	1.5	1/8
Robotic Nephrectomy	39	66.0	27.0	195.0	1/39	0/39	0/23	2.0	4/39
Robotic partial Nephrectomy	4	54.5	26.4	120.0	0/4	0/4	0/4	1.0	0/4
Robotic Nephro-ureterectomy	8	70.5	25.0	312.5	0/8	0/8	0/8	2.5	2/8
Robotic Pyeloplasty	10	43.0	24.0	140.0	0/10	0/10	NA	2.0	1/10
Robotic Adrenalectomy	1	73.0	25.0	100.0	0/1	0/1	0/1	2.0	0/1
Total	70	66.75	25.7	167.5	2/70 (2.9%)	0/70 (0%)	1/44 (2.3%)	2.0	8/70 (11.4%)

One positive margin was observed in a robotic prostatectomy patient (12.5%). Early continence (safety pad or less at 3 months) was achieved in 4 of 8 (50%) after prostatectomy. Eight patients (26.7%) had complications within 30 days, three were Clavien–Dindo class 3 and one class 4. Blood transfusion was not required in any of the cases.

## Discussion

This modified IDEAL Stage 2b evaluation shows that Versius has been integrated safely into multiple hospitals in the UK, that have established robotic surgery training and programs.

Surgical expertise and technical skills were not a factor in outcomes. Surgeons involved in the study, continued to perform surgeries with the Da Vinci robot, and performed dry simulation exercises with the Versius platform to assist with familiarity and orientation. This did not affect oncologic outcomes with only 1 patient having a positive margin from the robotic prostatectomy cohort and zero positive margins in the partial nephrectomy group. Median length of stay was not prolonged compared to other robotic platforms.

The total console time was relatively long, which is attributed to the learning curve at all sites. Furthermore, the times also reflect more challenging dissections, rather than issues with the use of the Versius platform. The prolonged console time did not affect the clinical safety and the outcomes. As discussed in the phase 1 study, positioning the robotic arms improved rapidly in each case. There were early issues related to clashing and it was difficult in the early series to utilize 4 arms for performing a robotic radical prostatectomy. It is anticipated however that with more cases, this will continue to improve.

With regard to complications, there were two intra-operative complications encountered in this study. Device failure occurred in one of the cases requiring conversion to laparoscopy. The other was a splenic bleed during nephrectomy requiring conversion to open. Even though the numbers in this study are small, this is similar to quoted rates of device failure with Da Vinci and HUGO. Furthermore, the nephrectomy requiring conversion to open is similar to that in literature, with some reporting rates as high as 13.2%. This was due to difficult dissection, rather than technical failure or surgical expertise. Post-operative 30-day complications were also consistent with known rates. There was no mortality, and no blood transfusions required in any of the cases. Similar findings were present in the study carried out by Hussain et al. at the Sindh Institute of Urology and Transplantation [11]. The authors also came across device failure in 2 out of the 106 urological cases performed which required replacement of the malfunctioning arm without the need to convert to laparoscopy or an open approach.

Across five hospitals, a range of common urological robotic procedures were shown to be feasible with Versius in this study. We acknowledge that cases were carefully selected as reflected by median age and BMI. However, as noted in our phase I study, lower BMI was paradoxically associated with more challenging surgery when compared to higher BMIs. Working with the engineering team at CMR, the problem was attributed to the Versius RSS software which deemed that the port pivot point had to be 5 cm from the point of insertion. This has since been corrected, meaning that this is no longer an issue.

Apart from a longer median console time, other outcomes recorded show that Versius RSS can perform these cases safely, with similar or lower rates of complications. We anticipate the median console time to reduce with improved understanding of port placement, and increased exposure and experience by surgeon and the theater team. This has already been reflected in reduced set-up and docking times with each case. It is important to note that we cannot derive too much with regard to learning curves as multiple surgeons were included in the study with a wide variety of procedures analyzed, each with its own varying learning curve. The inclusion of the mix of procedures was more for the purposes of deciphering whether or the Versius platform can be applied to multiple pathologies such as those existing robotic platforms.

A limitation of this study is of course that the number of cases was limited and therefore a statistically significant conclusion cannot be drawn from the data. However, it does provide some early evidence for the effectiveness of the Versius platform. Randomized controlled trials in the form of IDEAL stage 3 studies are required to form a more conclusive statement.

## Conclusion

A range of urological procedures were shown to be feasible and safe using the Versius across multiple sites.

## Data Availability

No datasets were generated or analysed during the current study.
